# Assessing the Ability of an Online Education Program to Modify Patient Expectations of Total Knee Arthroplasty Outcomes: Protocol for a Randomized Controlled Trial

**DOI:** 10.2196/71898

**Published:** 2025-10-30

**Authors:** Karen Ribbons, Frederick Rohan Walker, Michael Pollack, Michael Nilsson

**Affiliations:** 1 Hunter Medical Research Institute, Newcastle, Australia; 2 Callaghan Campus, College of Health, Medicine and Wellbeing, University of Newcastle Australia, Newcastle, Australia; 3 Centre for Rehab Innovations, University of Newcastle Australia, HMRI, Kookaburra Circuit, New Lambton Heights, Newcastle, 2305, Australia, 61 0240420758; 4 John Hunter Hospital, Hunter New England Local Health District, Newcastle, Australia; 5 Rehabilitation Research Institute Singapore (RRIS), Singapore, Singapore

**Keywords:** health literacy, patient education, expectations, total knee arthroplasty, musculoskeletal

## Abstract

**Background:**

Patient satisfaction with total knee arthroplasty (TKA) is strongly influenced by alignment between expectations and outcomes. Despite a satisfaction rate of 80%‐90%, dissatisfaction affects 10%‐20% of patients and is expected to grow with the increasing volume of TKA procedures globally. Misaligned expectations, often driven by unrealistic or overly optimistic recovery views, can lead to unmet goals, dissatisfaction, and unnecessary health care usage. Addressing these gaps through improved presurgical education has the potential for enhancing patient satisfaction, optimizing outcomes, and reducing the burden on the health care system.

**Objective:**

The main objectives of this study are to develop and evaluate a presurgical educational program, which focuses on patient expectations of surgical outcomes and facilitates patients setting realistic postsurgical goals. We will also assess the ability of the program to modify patient expectations, impact expectation fulfillment, improve satisfaction with postsurgical outcomes, and impact patient health literacy.

**Methods:**

A targeted education program will be developed in consultation with key stakeholder groups, including consumer advocates, orthopedic surgeons, health care providers, and physiotherapy and rehabilitation specialists, to address realistic patient expectations of TKA outcomes. Alpha testing with consumers will provide insights into the appropriateness of the program being developed. The ability of the program to modify patient expectation will be assessed in a longitudinal, parallel group, 2-armed randomized controlled trial involving 150 patients identified by their orthopedic surgeon as requiring TKA. Randomly allocated participants will take part in the education program within 5 weeks prior to their scheduled TKA (intervention group) or will be allocated to standard preoperative education (control group). The primary outcome will be a change in the Hospital for Special Surgery Total Knee Replacement Expectations Survey–transformed score measured prior to and following the intervention. At 6 months following TKA, expectation fulfillment and overall satisfaction will be measured. Inferential statistics will be used to test for differences in, or associations between, outcome measures within and between study arms. The methods appropriate to both dependent and independent samples will be used, including nonparametric methods for data in violation of normality and variance assumptions.

**Results:**

The education program will be developed from January to September 2025. The randomized trial will run from October 2025 to March 2027, with data analysis completed by April 2027 and results published in peer-reviewed journals by September 2027.

**Conclusions:**

This study will provide evidence on the effectiveness of a novel presurgical educational program in shaping patient expectations, promoting realistic goal setting, and improving TKA satisfaction. Findings will inform strategies to improve TKA patient care, health literacy, and satisfaction, potentially reducing dissatisfaction and associated burden on health care.

## Introduction

### Background

Total knee arthroplasty (TKA) is a well-established intervention for end-stage osteoarthritis, offering significant improvements in joint function and pain reduction, with low rates of revision and mortality [1,2]. While these clinical outcomes are critical from a health care provider’s perspective, they do not fully encompass the range of outcomes that matter most to patients, particularly those related to functional recovery and goal attainment following TKA [3-5].

Systematic reviews consistently report that 10%‐20% of patients remain dissatisfied with surgical outcomes, often due to persistent pain, limited range of motion, or insufficient improvement in knee function [6-9]. This dissatisfaction persists despite advances in prosthetic design [10], robotic-assisted surgical techniques, and improved implant alignment [11]. Importantly, dissatisfied patients incur up to 57% higher societal costs, including out-of-pocket therapies, lost productivity, and caregiver expenses postsurgery compared to satisfied patients [12].

With the number of TKA procedures projected to rise substantially over the next 2 decades [13,14], improving patient satisfaction is imperative, not only for individual outcomes but also for broader economic and health care system impacts.

Satisfaction with surgical outcomes is a multifaceted construct influenced by various factors [15], including disease severity, preoperative pain, and functional limitations [10,16,17], as well as psychological factors, such as mood and mental health status [18-20]. In addition, fulfillment of TKA patient expectations regarding postsurgical outcomes has been associated with satisfaction levels [7,21-23]. Patient expectations also predict residual pain and functional outcomes, underscoring the importance of goal setting [24,25]. However, unrealistic expectations may lead to discouragement and poor adherence to rehabilitation, while overly modest expectations may reduce motivation [25].

Social, demographic, and cultural factors also influence the nature and scope of patient expectations [26-28]. A comparative study of patients in the United Kingdom, United States, and Australia revealed that while overall satisfaction rates were similar, Australian patients were more likely to expect improved function at 12 months post surgery and placed greater emphasis on recreational activities and unaided mobility [29]. Our recent research in an Australian cohort further demonstrated that the majority of patient expectations centered on high-impact activities, such as golf, cycling, long-distance walking, and gym-based exercise [30].

Despite the importance of patients setting realistic expectations to optimize expectation fulfillment [31-33], this issue is not routinely addressed in clinical practice, partly due to the lack of standardized methods for assessing expectations and their fulfillment. Some studies have focused narrowly on pain reduction and basic functional outcomes, omitting patient-specific goals [21]. Others have used predefined activity lists to gauge importance [34]. However, no gold-standard approach currently exists for characterizing and evaluating patient expectations from TKA.

Despite the lack of a standardized approach, several groups have suggested approaches to assess patient expectations. One approach involves patient-reported surveys that categorize expectations by importance preoperatively and assess fulfillment postoperatively [34,35]. This method allows for structured quantification of expectation levels and categories. A more recent alternative is inductive content analysis, which uses open-ended questions to capture patient-specific expectations without the constraints of predefined categories [36], which enables a more personalized understanding of patient expectations and their fulfillment.

While patient expectations can certainly be assessed, the more critical challenge lies in actively shaping those expectations so that patients are positioned to be highly engaged in their recovery and ultimately satisfied with their outcomes. Achieving this requires more than simple information sharing—it calls for deliberate, structured patient education. However, despite its importance, such education is often underemphasized or even absent in current presurgical discussions. Indeed, preoperative education aimed at modifying expectations has shown promise in enhancing patient satisfaction [35,37]. Such interventions help patients set realistic goals, leading to reduced expectation levels and improved fulfillment at 12 months post surgery [37]. In addition, targeted education has been shown to reduce the number of expectations compared to standard education alone [35]. Collectively, these findings support the integration of expectation management into preoperative care to optimize patient outcomes.

Musculoskeletal health literacy among patients seeking orthopedic care has been found to be lower than general health literacy [38]. While there is a substantial volume of online information regarding TKA, its utility is limited due to poor readability, understandability, and actionability [39]. This is concerning, as low health literacy is associated with patients with musculoskeletal conditions setting lower expectations for surgical outcomes [40-42].

Structured educational interventions have demonstrated efficacy in improving health literacy. Even a single structured session has been shown to enhance health literacy among patients with orthopedic conditions [43]. Furthermore, eHealth education has shown promise in improving both health literacy and self-efficacy in older adults [44]. Despite these encouraging findings, uptake of educational resources related to TKA rehabilitation and recovery remains limited [45].

Clinicians play a critical role in guiding patients toward realistic expectations by providing education on achievable outcomes while also understanding individual patient priorities. This dual approach supports realistic expectation-setting, shared decision-making, and a patient-centered model of care. With growing demand from health consumers to actively participate in decisions regarding their health care, there is a clear need for the development of targeted educational models and decision aids. TKA represents a pertinent example, given the high volume of procedures performed and the substantial proportion of patients who report residual dissatisfaction.

### Aims and Objectives

In the current project, we will collaboratively develop a novel educational program for patients undergoing TKA with orthopedic care teams, consumer advocates, and key health care provider stakeholders, which will focus on providing information that will inform and encourage patients to set realistic expectations of TKA surgical outcomes. The ability of the program to influence the type of expectations patients set prior to surgery will be assessed by comparing patients’ expectations before and after the program and by comparing expectations of those who participate in the program to those who receive “standard clinical care” alone. In addition, the project will determine if education program participation is associated with a higher level of expectation fulfillment, satisfaction with surgical outcomes, and improvement in health literacy. We will apply a number of different methods to define patients’ expectations, using both closed- and open-ended questionnaires to evaluate the scope of responses achieved by each approach.

## Methods

### Study Design

#### Study Stages

The study will take place over 2 stages. Stage 1 will focus on the collaborative design of an education program intervention to be delivered to patients prior to their TKA, while Stage 2 will involve a randomized controlled trial, which will determine if the developed education intervention is effective in modifying patient expectations regarding TKA outcomes and assess the impact of the intervention on expectation fulfillment and overall satisfaction with surgical outcomes and health literacy 6 months following the TKA.

#### Stage 1

##### Overview

The educational materials to be developed will consist of multiple videos that patients will access online. Each video developed will run for no more than 5 minutes and address different aspects of recovery and capacity to undertake a range of different tasks and activities following TKA surgery.

##### Consultative Process

The content of the videos will be developed through consultancy with key stakeholder groups, including orthopedic teams (local orthopedic surgeons who regularly undertake TKAs and rehabilitation specialists), consumer advocates (people who have undergone a total knee replacement within the last 5 years), and the study steering committee (including members of the research team and collaborating researchers with experience in behavior change methodology and knee replacement rehabilitation). We will approach orthopedic surgeons and rehab physicians from the 3 main private hospitals who undertake TKAs in the greater Newcastle New South Wales area to be involved in our clinical consulting committee. From our recently conducted SuPeR Knee: Support, Predict, Recover study [46], we will send email requests to participants who had consented to be made aware of future research activities being undertaken by our team and ask them to complete an expression of interest form. Interested people will be asked to provide details of their age, sex, date of knee replacement, employment details, and their reasons for wanting to take part. Five advocates will be selected, giving a diverse range of individuals with varied experiences. The key stakeholder groups will be advising on how to present material that can convey to the viewer the importance of setting realistic expectations of outcomes following a TKA. A combination of mixed media styles will be applied, including presentations by rehabilitation specialists and interviews with patients who have lived experience of life following TKA, as well as other graphic tools, such as real-life scenarios and text to clarify key concepts.

##### Video Design Principles

The design of the educational videos will be guided by social cognitive theory (SCT) [47] and the Information-Motivation-Behavioral Skills model [48], both of which are foundational frameworks for promoting health behavior change. SCT posits that individuals’ ability to make choices is shaped by the dynamic interaction of cognitive, behavioral, and environmental factors [47]. It also highlights the importance of observational learning, which can be enhanced by factors such as attention and engagement. To support this, our videos will present relatable content by addressing common themes around expectations identified by individuals preparing for total knee replacement [34]. These themes will reflect variations in medical status, lifestyle, personal priorities, and belief systems. To reinforce the central learning objectives, each video will reiterate key concepts related to expectation-setting and goal-directed behavior. Within SCT, self-efficacy plays a central role in learning and behavior change [47]. Accordingly, the videos will showcase a range of activities and tasks that individuals may aspire to undertake post surgery. These examples are intended to foster viewer engagement and encourage personal reflection on lifestyle, capacity, and realistic goal setting. To enhance relatability, the videos will incorporate plain language, open-ended questions, and visuals of real-world settings, including perspectives from patients with lived experience of TKA. Institutional branding will be used to establish credibility, and narration will be provided by qualified physical therapists and exercise physiologists, offering a trusted and authoritative voice [49].

Content will also be informed by principles from the Science of Learning and the Science of Instruction in Multimedia [50]. Using videos as the media of choice enables the integration of visual and auditory cues aimed at engaging working memory and facilitating the integration of new information with existing beliefs stored in long-term memory. This offers the potential for enhancing the retention of information being conveyed and encouraging behavior change, which extends beyond that which could be achieved by providing printed materials.

Using a constructivist learning model, the material presented will go beyond biomedical information to encourage viewers to actively consider their own lifestyle, values, and realistic postsurgical outcomes [51].

Key elements of behavior change incorporated into the information will include enhancing psychological capability and reflective motivation through the provision of knowledge and understanding about recovery following TKA [48,52]. Reflective motivation will be further supported by eliciting positive emotions associated with setting achievable goals, as well as highlighting the potential negative consequences of unrealistic expectations.

##### Alpha Testing

Our consultative process will involve the development of scripting and storyboards for videos with each of our stakeholder groups. Materials will be reviewed until a consensus regarding the content and format of the educational materials among the consulting groups has been reached. Subsequently, videography and initial editing will then be undertaken, and the first prototype materials generated. Prior to generating the final version of the videos, we will undertake alpha testing by seeking input from an additional consumer group who have undergone a recent total knee replacement. The alpha testing will constitute an evaluation of the understandability (content scope, word choice and style, organization, layout, and design) and actionability of the prototype. The feedback provided will be used to make improvements in the material leading to the generation of the final version.

Initially, we will enroll 5 participants to be involved in the alpha testing. Purposive sampling will be undertaken based on sex and age to maximize the range of viewpoints derived. Participants will be recruited from the cohort who participated in our recently conducted SuPeR Knee: Support, Predict, Recover study [46]. Consequently, they have had prior familiarity with interviewer KR, who was the project manager in this study. Participants will be asked to provide the research team with details regarding their knee replacement history, the highest level of education achieved, information regarding their current health conditions, and the completed 12-item European Health Literacy Survey Questionnaire (HLS-EU-Q12) [53]. A pragmatic, mixed methods approach will be adopted to capture feedback regarding the educational videos and guide further editing. We intend to adopt a qualitative descriptive approach to enable us to gain firsthand knowledge from the patient group regarding the content of our prototype [54,55].

After viewing each video, participant feedback will be provided by completion of the Patient Education Materials Assessment Tool—Audio Visual (PEMAT-A/V) [56,57], which addresses the relevance (content scope), understandability (word choice and style, layout, and design), and actionability (intended actions for the viewer to take) of the materials presented. To provide further insights, qualitative data will also be collected to verify the survey responses.

Participants will be contacted to arrange a time for a one-on-one structured interview with a member of the research team to discuss their responses to the PEMAT-A/V surveys and provide additional feedback regarding the educational program they viewed. To provide credibility to the research data, interviews will be undertaken by members of the research team who have extensive experience in undertaking qualitative research interviews and data analysis (KR, female, PhD, study chief investigator, or LS, female, BComm, BPsych, study research assistant). Interviews will last for approximately 30 minutes, with participants having the option for this to be undertaken as a recorded face-to-face meeting, an online Zoom (Zoom Video Communications) video call, or a phone call. Sessions will be recorded, and transcripts viewed by the participants to verify that they reflect their statements. The following open-ended questions will be used to guide the discussion to elucidate information regarding the participants’ experiences with the material they were presented:

1. Can you explain why you gave each score on the PEMAT-AV survey, and are there any changes we need to consider?
2. Do you trust the information that was provided?
3. Do you think that the information provided will influence patients’ decisions about what they can expect following a TKA?

The focus of the interviews will be based on the participant’s responses to the PEMAT-AV surveys and consequently focus on their narrative, with the interviewer providing additional questions to further expand the participant’s views, providing confirmability of the findings. Both researchers undertaking the interviews will also be involved in the development of the education materials, with an overarching aim to develop high-quality educational material in this study. Regarding reflexivity, both researchers will be aware of their own viewpoints regarding the materials being reviewed but keep the narrative of the participant as the focus of the discussion to reduce interviewer biases to influence the topics discussed [58]. To assist with the transferability of the data collected, interviewers will provide a written account for each interview describing additional participant characteristics, including cultural and social features that may have impacted the interview dynamic.

### Stage 2

The impact of educational materials generated in stage 1 will be evaluated in a longitudinal, parallel group, 2-armed, block randomization trial with an allocation ratio of 1:1 [59]. Patients undergoing TKA will be enrolled when they have been scheduled for the surgical procedure and will be involved in study assessments for a total of 32 weeks (8 months) from study entry. Referrals of potential study participants will be provided by the participating orthopedic surgeons when their patient has consented to undergo a TKA. The maximum wait time between patient consent and surgery taking place is 6 weeks. Consequently, all presurgery assessments and the study intervention will take place within this 6-week window. Following study consent, baseline assessments will be undertaken at study entry, which is likely to be 5 weeks prior to TKA. The intervention (viewing the educational videos+standard care) or control (standard care alone) will be delivered 4 weeks prior to the scheduled TKA. Standard care will encompass any educational materials provided to the patient by their surgeon and health care provider. For the intervention group, participants will be provided online access to the educational videos. They will have the capacity to view the videos several times if they desire and will record the number of times each one is viewed. Postintervention assessments will be conducted 3 weeks prior to TKA, and postsurgical outcome measures will be undertaken at 6 months following the TKA.

### Study Setting

The stage 2 randomized trial will be run as a single-center study, centrally managed by the Centre for Rehab Innovations, University of Newcastle, New South Wales. We intend to recruit patients who are scheduled for TKA to take place at private health care facilities within the Greater Hunter Region, New South Wales, Australia and is likely to include, but is not limited to, Lake Macquarie Private Hospital, Gateshead; Maitland Private Hospital, East Maitland; and Lingard Private Hospital, Merewether, New South Wales.

### Study Population and Eligibility

#### Stage 1

We will be sourcing potential study participants from the patient cohort who recently took part in the SuPeR Knee: Support, Predict, Recover study [46]. Study participants who consented to be contacted will be identified and informed about this study. We will recruit 5 patients who have already undergone TKA surgery to assist with alpha testing of the education program being developed in this stage of the study. Patients will need to meet the inclusion and exclusion criteria listed in Textbox 1.

Textbox 1. **Inclusion and exclusion criteria for stage 1.**

**Inclusion criteria**

- Have undergone a primary total knee arthroplasty (unilateral or bilateral) within the last 5 years
- Adult patients (>18 years)
- Have the capacity to complete a series of self-reported questionnaires (presented in English)
- Are able to provide informed consent

**Exclusion criteria**

- Have undergone a recent primary total knee replacement due to acute trauma
- Are unable to provide total knee arthroplasty consent

#### Stage 2

We will recruit 150 patients who have been identified as requiring a TKA to take part in the randomized controlled trial being undertaken in this stage of the study. Patients will need to meet the inclusion and exclusion criteria listed in Textbox 2.

Textbox 2. **Inclusion and exclusion criteria for stage 2.**

**Inclusion criteria**

- Have been identified as requiring a primary total knee arthroplasty (unilateral or bilateral) by their treating orthopedic specialist
- Adult patients (>18 years)
- Have the capacity to complete a series of self-reported questionnaires (presented in English)

**Exclusion criteria**

- Will be undergoing a primary total knee replacement due to acute trauma
- Have undergone previous knee replacement surgery
- Have had knee surgery within the preceding 6 months, have knee surgery scheduled within the next 6 months or are undergoing a revision for existing joint surgery
- Are unable to provide informed consent

### Recruitment and Enrollment Procedures

Eligible participants will be contacted by a member of the research team. This will be a follow-up phone call in stage 1, after information has been posted to them; and in stage 2, after they have received the patient information sheet and study information flyer from their surgeon. For stage 2, participants will be recruited from referrals from orthopedic surgeons who undertake total knee replacement procedures at Lake Macquarie Private Hospital, Gateshead, New South Wales, Australia; Lingard Private Hospital, Merewether, New South Wales, Australia; or Maitland Private Hospital, East Maitland, New South Wales. The surgeon and/or their staff will (1) ensure every eligible patient receives a patient information sheet and a flyer, (2) direct patients to contact information for the research team on the patient information sheet/flyer, and (3) share a list of each patient receiving the patient information sheet with the study team. A member of the study team will follow up with each eligible patient via telephone to ensure that they have understood the information they received and to determine if they are interested in participating in the study.

A member of the study team will follow up with each eligible patient via telephone to (1) ensure that they have understood the information they received and (2) determine if they are interested in participating in the study.

Eligible participants can choose not to participate when they receive the patient information sheet and study information flyer. If they do decline to participate, they will not be contacted further. The research team member will ask if the participant has any questions about the study and if they are interested in participating.

If patients opt to have their study questionnaires accessed via the study online platform, they will be provided a copy of the patient information sheet when they access this system via a participant-specific login. Following review of the patient information sheet, they will be asked to acknowledge their consent to participate in the study and provide an electronic consent on the electronic letter of consent. Questionnaire templates will not be accessible to the participants until the consent field on this form has been completed.

If patients opt to have their study questionnaires posted to them, a copy of the patient information sheet and informed consent form will be included in the posted package. Participants will be asked to sign the consent form prior to commencing completion of the questionnaires. The completed consent form and questionnaires will be posted back to the research team.

Participants can decline to participate at any time point during the study, which will not impact their medical care. If patients decide to withdraw from the study, they can specify if their data is to be removed from the study or if they are willing for it to be used as part of the analysis.

### Sample Size

#### Sample Size Estimation and Justification

##### Stage 1

We will initially involve 5 individuals who have undergone a TKA within the last 5 years to take part in video alpha testing encompassing the understandability and actionability of the prototype of the educational videos. Content analysis will be via an inductive approach [60]. If we find that the issues identified in the prototype videos differ substantially between each participant and saturation of the feedback content is not occurring, we will enroll 5 further individuals to undertake the alpha testing, such that feedback from up to 10 individuals will be included in stage 1. The purposive sampling approach being adopted will enable a diverse group of viewpoints to be derived. Taken together with the collaborative approach between the stakeholder groups involved in the design component of this study, we are confident that the number of participants involved in the alpha testing is sufficient to support the development of education materials to be further evaluated in stage 2 of this study.

##### Stage 2

A sample of 150 individuals who have been identified as requiring a TKA by their treating orthopedic specialist (2 subgroups of n=75) will be recruited. This will allow for expected dropouts (approximately 22/150, 15%) and ensure a minimum sample size of 64 for each group, which will enable the study to be adequately powered (see calculations below). A screening log will be kept and will collate reasons why any participants were screened but did not take part in the study.

### Power Calculation: Sample Size Estimate

The number of participants was selected based on previously published findings using the Hospital for Special Surgery Total Knee Replacement Expectations Survey (HSS-TKR) as our primary outcome measure to demonstrate a clinically meaningful change in the overall patient expectation score [35]. Mancuso et al [35] describe a clinically meaningful change in expectations to be associated with a 6-point change in transformed HSS-TKR total scores. To determine a significant difference between 2 groups of patients, such that α=.05, power 80%, and SD 12, will require 64 patients per group. In this study, we will enroll 75 patients per treatment arm to allow for any potential loss of participation at the follow-up time points.

### Randomization Procedures

#### Block Randomization

To ensure a balance in the sample size for each treatment arm during the participant recruitment phase, we will apply block randomization [59]. For a total of 150 recruited participants, we will generate 38 blocks of 4 participants per block with a 1:1 allocation ratio between the intervention and control arms. The randomization list and block allocation details will be generated using software provided by Sealed Envelope Ltd (2022)[] .

#### Stratification

We recognize that the level of standard clinical care, with respect to the scope and format of educational materials provided to patients undergoing TKA, may vary significantly between health care providers represented in the recruitment sites involved in the study, and this may influence expectations set by participants. In light of this, we will include “health care provider” as a stratification variable.

#### Replacements

Replacements will be enlisted if participants need to withdraw prior to their TKA taking place. To maintain the randomization ratio of 1:1, the replacement would be allocated to the same treatment arm of the withdrawn participant.

### Data Collection Tools and Procedures

#### Online Data Collection and Centralized Data Repository

##### Screening logs

Screening logs will be maintained for all potential candidates being recruited to the study and will contain identifiable patient information. The file will be stored online as a password-protected file on the secure University of Newcastle server.

##### Deidentification of Patient Information

All consented participants will be allocated a study code comprising a patient ID number and their initials. All data collected in the study will be identifiable by the study code and remain deidentified during the data collection and analysis phases of the study.

##### Data Collection Platform

Study data will be collected and managed using the REDCap (Research Electronic Data Capture; Vanderbilt University) tools hosted at the Hunter Medical Research Institute [61,62]. REDCap is a secure, web-based software platform designed to support data capture for research studies, providing (1) an intuitive interface for validated data capture, (2) audit trails for tracking data manipulation and export procedures, (3) automated export procedures for seamless data downloads to common statistical packages, and (4) procedures for data integration and interoperability with external sources.

REDCap study-specific access will be established, allowing secure access by the research team with all assessment tools formatted as online forms to enable direct data entry by the study participants. Participants will be sent an email from REDCap containing an encrypted link to their forms for completion. If participants request to complete hard copies of forms, these will be generated and posted to the participant by the study team, with the completed form data subsequently entered into the REDCap platform by a member of the study team.

Forms will be disseminated for completion at 3 distinct time points–prior to their TKA at study entry (baseline), following the education intervention, and 6 months following their TKA, as shown in Table 1.

**Table 1.** Assessments to be undertaken in the randomized controlled trial and frequency of collection.

Measure (tools)		Study time points		
		Baseline	Post intervention	6 months post TKA^a^
Clinical and demographic details				
Patient-reported age, sex, highest level of education achieved, employment status, and diagnosis		✓		
Comorbidities				
Charlson Comorbidity Index [63]		✓		✓
Knee symptomology				
12-item short-form for the Knee Injury and Osteoarthritis Outcome Score [64]		✓		✓
Oxford Knee Score [65]		✓		✓
Forgotten Joint Score [66]				✓
Mood status (depression, anxiety, and stress)				
21-item Depression, Anxiety and Stress Scale [67,68]		✓		✓
Quality of life				
EQ-5D-5L [69]		✓		✓
Health literacy				
12-item European Health Literacy Survey Questionnaire [53]		✓		✓
Expectations				
Hospital for Special Surgery Total Knee Replacement Expectations Survey [34]		✓	✓	
Direct Questioning of Objectives Questionnaire Index [70]		✓	✓	
Activity expectations [30]		✓	✓	
Expectation fulfillment				
Hospital for Special Surgery Total Knee Replacement Expectations Survey				✓
Direct Questioning of Objectives Questionnaire				✓
Activity expectation satisfaction				✓
Satisfaction with outcomes				✓

a TKA: total knee arthroplasty.

### Study Questionnaires

#### Comorbidities

We will use the Charlson Comorbidity Index, which provides a weighting to concomitant health conditions based on the nature of the condition and age of the individual and is used to predict 1-year mortality. The higher the score, the higher the mortality risk. We will apply the same 10 medical conditions and weightings used by Chaudry et al [63]. Participants will be asked to indicate if they experienced any of the 10 medical conditions provided, at study entry and at 6 months after TKA.

#### Knee Symptomology and Function

Symptomology of the affected knee that will undergo TKA will be assessed using the 12-item version of the Knee Injury and Osteoarthritis Outcome Score (KOOS-12) [64]. KOOS-12 is a 12-item measure derived from the original 42-item KOOS. KOOS-12 contains 4 KOOS pain items, 4 KOOS function (activities of daily living and sport/recreation) items, and 4 KOOS quality of life. Each item is rated using a 5-point Likert scale from 0 to 4, left to right, with 0 representing no knee problems and 4 representing extreme knee problems [64]. Items with lower scores are indicative of lesser symptoms, and higher scores reflect worse symptomology. Participants will be asked to complete a copy of this questionnaire at study entry and at 6 months after their surgery.

We will also apply the Oxford Knee Score (OKS) [65]. The OKS is a patient-reported outcome measure that consists of 12 questions about an individual’s level of function, activities of daily living, and how they have been affected by pain over the preceding 4 weeks, before and after a total knee replacement. Each item is rated using a 5-point Likert scale from 0 to 4, with 0 representing no knee problems and 4 representing extreme knee problems [65]. Items with lower scores are indicative of lesser symptoms, and higher scores reflect worse symptomology. In this study, the OKS will be applied at study entry and at 6 months after their surgery.

At 6 months following TKA, we will apply the Forgotten Joint Score (FJS) [66]. The FJS is a 12-item questionnaire concerning the participants’ awareness of their artificial joint during activities of daily life [66]. For each question, the participant can choose between 6 response options, namely never (0 points), almost never (1 point), seldom (2 points), sometimes (3 points), and mostly (4 points). The summation of the scores is then divided by the number of items responded to. The mean score is multiplied by 25 to give a score from 0 to 100, which is then subtracted from 100. Larger scores are indicative of greater “forgetting” of the artificial joint.

#### Mood Status

The mood status of our study participants will be assessed using the 21-item Depression Anxiety Stress Scales (DASS-21) [67]. The full DASS and abbreviated DASS-21 have been validated to measure the 3 negative emotional states (ie, depression, anxiety, and stress) in research and clinical settings [68]. Each of the DASS-21 subscales contains 7 items. Participants will be asked to use 4-point severity/frequency scales to rate the extent to which they have experienced each state over the past week. Scores for depression, anxiety, and stress will be calculated by summing the scores for the relevant items. Higher scores are indicative of higher levels of symptoms. All scores derived from the 21-point scale will be multiplied by 2 to enable comparison to the full 42-point DASS and determine clinical cutoffs for symptom severity. In this study, participants will be asked to complete the DASS-21 questionnaire at study entry (prior to their TKA) and at 6 months after their surgery.

#### Quality of Life

The EQ-5D-5L was developed to describe and value health status across a wide range of disease areas [69]. The tool contains a short descriptive system of the respondent’s health status and a visual analog scale to summarize health status (EQ-VAS). The descriptive system contains 5 dimensions, including mobility, self-care, usual activities, pain/discomfort, and anxiety and depression. Participants will choose the most appropriate severity of each dimension on a 5-point scale ranging from “no problems” to “unable to / extreme problems. A summary EQ-5D index score will be derived using dimension weightings from recent value sets described for the Australian population [71]. The EQ-VAS provides an overall current health rating score ranging from 100 (the best health) to 0 (the worst health). In this study, participants will be asked to complete the EQ-5D-5L questionnaire at study entry (prior to their TKA) and at 6 months after their surgery.

#### Health Literacy

Health literacy is defined as the degree to which individuals can obtain, process, understand, and communicate about health-related information needed to make informed health decisions [72]. In this study, we will assess participants’ health literacy using the shortened version of the HLS-EU-Q12 [53]. Participants will be presented with a number of competencies encompassing information relating to health care, disease prevention, and health promotion and covering 4 cognitive domains, including proficiency in accessing, understanding, appraising, and applying health information. Respondents will rate the level of difficulty in undertaking each competency on a 4-point scale ranging from 1 (very difficult) to 4 (very easy), with higher total scores indicative of higher health literacy. In this study, participants will be asked to complete the HLS-EU-Q12 at study entry (prior to their TKA) and at 6 months after their surgery.

#### Expectations

As there is no current gold standard for characterizing patient expectations of TKA outcomes, we will be adopting a mixed methods approach and using both standardized self-report, closed-response surveys and open-ended questions to quantify patient expectations and changes in these expectations over time. To evaluate the change in expectations, all participants will be asked to complete the following questionnaires at study entry and following the intervention.

#### Hospital for Special Surgery Total Knee Replacement Expectations Survey

The HSS-TKR [34] is a closed response survey, which enables respondents to provide ratings for the importance of 17 common patient expectations of outcomes following TKA, including pain, physical function, and psychological items. Importance ratings range from 1 (very important) to 5 (does not apply). The survey quantifies the level of importance of common knee replacement patient expectations and has been applied in numerous studies [73].

#### Direct Questioning of Objectives Questionnaire

Methods comparable to those applied by Karunaratne et al [70], using open-ended questions related to participant expectations, will be applied. Participants will be asked to list at least 1 and up to 5 of their most important expectations of TKA surgical outcomes, and then an assessment will be given for two domains for each expectation on 11-point numerical rating scales: (1) the importance of each expectation and (2) the current ability to achieve the task / item. The reported values for importance and ability will be interpreted as “low” if ≤5, and “high” if ≥8. A Direct Questioning of Objectives Questionnaire Index (DQO Index) will be derived from the product of the score for each domain.

#### Activity Expectations

We will use an open-ended question to identify participant expectations relating to their most important activities they want to undertake following TKA [30]. Participants will be asked to identify 3 activities important to them to achieve following the TKA by responding to the following question:

*What activities are you hoping to be able to do after your knee surgery that you cannot do now? (eg this could be an everyday life activity, employment or work activity, social activity, specific movement / motion or exercise etc.)*

#### Proceeding With TKA

Following exposure to the education program (intervention arm) or standard care (control arm), all participants will be asked if they will be proceeding with their TKA and, if so, to confirm the date of the procedure.

#### Expectation Fulfillment

The level of expectation fulfillment or satisfaction to undertake goals identified will be determined at 6 months after TKA for participants who proceeded with surgery.

For the HSS-TKR items, participants will be asked to indicate the extent of fulfillment of the expectations, identified prior to surgery, using a 5-point Likert scale, ranging from 5 (completely) to 1 (not at all).

The DQO Index will be repeated post TKA, and differences with values obtained prior to TKA will be indicative of the ability to achieve the task.

For activity expectations identified in the pre-TKA assessment, participants will be asked to rate how satisfied they were that each activity could be undertaken using a 7-point Likert scale ranging from 1 (completely dissatisfied) to 7 (completely satisfied).

#### Satisfaction

At 6 months after the TKA, all participants who underwent surgery will be asked to respond to the following:

*Overall, how would you describe the results of your operation?*

Participants will provide a response on a 5-point Likert scale, ranging from 1 (poor) to 5 (excellent).

#### Feedback Survey

A study participation evaluation survey will be included at the end of the study to enable participants to provide the research team with feedback regarding the intervention and assessments they were asked to undertake and the overall conduct of the study. It will also give study participants the opportunity to provide ideas for future areas of research they consider important to be addressed in recovery from TKA surgery. For those who did not proceed with undergoing a TKA, information will be collected regarding alternate treatment procedures they have undertaken.

### Primary Outcome

The primary outcome of the trial will be a change in patient postsurgical expectation score (within individual patients’ scores), derived from the HSS-TKR questionnaire, conducted prior to and following intervention exposure.

### Secondary Outcomes

The following secondary outcomes will also be evaluated:

1. The number of participants who decide not to proceed with their TKA.
2. The level of patient expectation fulfillment derived from the HSS-TKR questionnaire, the DQO Index, and activity expectations, conducted 6 months after the TKA
3. The level of patient satisfaction with surgical outcomes assessed at 6 months after the TKA.
4. A change in patient health literacy (within individual patients’ scores), derived from the HLS-EU-Q12, conducted at study entry and at 6 months after the TKA

### Data Analysis

#### Stage 1

A Method Triangulation approach will be taken in the alpha testing of the developed educational program, encompassing the participant responses to the PEMAT-A/V survey and content analysis of structured interviews [74]. This will inform editing or adjustments to the prototype videos to enable the final version to be generated and evaluated in stage 2.

##### PEMAT-AV Tool

Two subscores will be derived from the provided survey ratings, including the understandability and actionability scores. These scores will be provided as the total points (agreement with the statements provided) for each domain, expressed as a percentage of the total possible points. Higher scores will be indicative of good understandability and clarity of the actionability of the information provided in the videos. The scores and responses provided for each statement in the survey will be used to formulate the topics addressed in the subsequent interview between the participant and the researcher.

##### Content Analysis of Qualitative Data

The interviews will be conducted by either research team member (KR or LS) during a 30-minute, one-to-one session with the research participant. Each interviewer will review the transcript and validate the content, such that it reflects the PEMAT-AV scores for each video. The content concepts will be identified to reflect the acceptability of the prototype videos or potential changes or edits that need to be considered by the research team. To assist with ensuring the dependability of the data, the interviewers will then review the content analysis undertaken by the other interviewer and again undertake their own validation and identification of content concepts for each transcript. Both interviewers will then meet and compare each one’s content analysis and discuss until a consensus is reached. Coding will be undertaken such that classifiers will be applied to the common concepts identified across all transcripts. The interviewers will then determine if content saturation has been achieved or if additional participants will need to be recruited for the alpha testing.

### Stage 2

Descriptive and inferential statistical analyses will be undertaken to describe the characteristics of the participant cohort and to assess the research hypotheses, respectively. Clinical and demographic characteristics will be described using proportions, measures of central tendency (eg, mean and median), and dispersion (eg, SD, IQR). Inferential statistics will be used to test for differences in, or associations between, outcome measures within and between groups of interest. Methods appropriate to both dependent and independent samples (eg, *t* test, ANOVA, and correlation) will be used, including nonparametric methods for data in violation of normality and variance assumptions (eg, Mann-Whitney *U* and Kruskal-Wallis). The threshold for statistical significance will be set at a *P* value of .05.

To determine the impact of the health education program to change expectations, the primary outcome measure (HSS-TKR) will be measured and compared between the 2 treatment arms. The within-patient changes of the open-ended measures of patient expectations between T_0_ and T2 will also be compared between the 2 treatment arms to further inform the efficacy of the education platform intervention. We will use data from all participants post surgery to determine the association between expectation fulfillment and overall satisfaction. Activity expectation fulfillment satisfaction and overall procedure satisfaction generate ordinal datasets with associations between the 2 measures undertaken using Spearman rank correlation coefficients.

### Ethical Considerations

#### Human Subject Ethics Review Approval

This research methodology was peer-reviewed by the Hunter Medical Research Institute-nib grant committee and approved by the School of Medicine and Public Health at the University of Newcastle, New South Wales, Australia, in accordance with the Australian Code for the Responsible Conduct of Research. The study will be conducted according to the National Statement on Ethical Conduct in Human Research (2023). Ethical approval was granted by the University of Newcastle Human Research Ethics Committee (approval ID H-2024‐0312) on January 7, 2025.

#### Informed Consent

All participants taking part in each stage of the study will provide written or electronic consent prior to undertaking any study-related activities. Participant involvement is completely voluntary, and they can choose to withdraw from the study at any time. If they decide to withdraw, they will be asked to notify the research team. The participant will be asked if they consent for any data already collected from them during their study involvement to be used by the research team in their data analysis, or if they prefer that on withdrawal all collected data be destroyed and no longer be accessible for analysis. They will also be asked to provide a reason for their withdrawal, but a response is not mandatory.

#### Privacy and Confidentiality

To enable participants to be contacted, identifiable contact details will be made available to the research team. All data collected and analyzed in both stages of the study will be deidentified. Patient data will be linked between different time points using a unique study code identifier allocated to each participant at the time of study enrollment. Master participant identification decoding records will be maintained and accessible by the chief investigator.

#### Compensation Details

All participants will take part in the study on a voluntary basis with no financial compensation provided.

#### Participant Identification

The deidentified data will be analyzed and presented in publications in scientific journals and at international/national conferences. All data will be presented at a group level, which means that no individual person will be able to be identified in any reports that are generated about the project. Individual participants will not be identifiable in any of the outputs generated from the research project, but individual anonymous responses may be quoted. Nonidentifiable data may be shared with other parties as part of a peer-review process to verify the robustness and integrity of the study or to contribute to further research and public knowledge.

## Results

This study was funded by a grant from the Hunter Medical Research Institute provided by nib insurance in December 2024. We forecast enrolling participants to take part in the alpha testing of the education materials developed in stage 1 of the study in October 2025 to enable finalization of the education program in November 2025. Recruitment to the stage 2 randomized trial designed to evaluate the novel educational materials will take place from December 2025 to September 2026. Final results are expected by April 2027.

## Discussion

### Expected Outcomes

This study responds to the unmet clinical need to address the issue of improving the level of satisfaction experienced by patients following TKA surgery. With the anticipated significant increase in the number of these surgical procedures being performed annually over the next two decades, it is important to consider improving outcomes for a significant number of patients who potentially may experience outcomes that do not meet their expectations following TKA. As the level of satisfaction with surgical outcomes has been shown to be influenced by unmet goals and high expectations of outcomes set prior to surgery, our focus was to enhance the ability for patients to set expectations, which were realistically likely to be met and thereby improve their satisfaction with the outcomes achieved.

We will be engaging in a collaborative consultation process with key stakeholder groups, including consumer advocates and clinical and service provision staff, to support the development of meaningful and accessible education materials that will encourage patients undergoing TKA to set goals that are realistic. The efficacy of the developed educational program will be evaluated in its capacity to impact the expectations of patients undergoing TKA prior to surgery and the capacity to influence postsurgical expectation fulfillment, outcome satisfaction, and level of health literacy. The findings will inform future implementation strategies of the wider application of the educational materials developed.

### Comparison With Prior Work

The approach adopted in this protocol builds upon existing research that has demonstrated the impact of unfulfilled expectations on patient satisfaction with outcomes following TKA. Previous studies have also indicated that Australian patients undergoing TKA set high expectations regarding TKA outcomes, with goals focused on achieving high physical impact pursuits following surgery [29,30]. While several studies have demonstrated the capacity of presurgical education interventions influencing patient expectations regarding TKA outcomes [35,37,75], widespread use of educational materials focusing on setting realistic expectations regarding surgical outcomes has not been adopted. This may, in part, be due to limitations in accurately characterizing patient expectations due to a lack of gold standards to capture this information. There is also a lack of quality educational materials being developed and distributed to patients undergoing TKA [76,77]. To address these issues, we will be using a multimodal approach to characterize patient expectations and expectation fulfillment by applying closed- and open-ended self-report tools to enhance the accuracy of identifying individual patient expectations and goals. We will also be using quantitative and qualitative methods in the alpha testing of the education materials developed with a TKA consumer cohort, which will enhance the quality of the materials being developed, and we will consider these findings to optimize the suitability of the final educational program to be applied. In our study, we will also be determining if our presurgical videos can impact participant health literacy. In a comparison of the influence of a single video presentation regarding osteoarthritis and recovery following total joint replacement in patients with orthopedic conditions compared to that obtained following a preoperative face-to-face consultation with their clinician [78], not only did the video significantly increase patient health literacy, but the level of knowledge improvement was greater than that achieved by those who underwent a preclinical consultation only.

### Potential Impact and Clinical Implementation

If successful, the educational program developed in this study has the potential to impact TKA patient satisfaction with surgical outcomes through improvement of fulfillment of more realistic expectations being set prior to surgery. This will support a more patient-centric classification of surgical “success,” which extends beyond clinical parameters. Improved satisfaction may also reduce usage of health resources postoperatively. The findings will support a broader implementation strategy for a widespread application of the education materials in the clinical setting, which could encompass patients in both the private and public sectors, with the potential to improve patient health literacy and satisfaction with TKA on a large scale. Findings from this study may also influence preoperative processes for other orthopedic surgeries, including total hip replacement and other elective surgical procedures.

### Strengths and Limitations

The strength of the design of the educational materials being developed and tested in this study is the collaborative approach we will be adopting through the involvement of key stakeholders including clinical, research, and consumer groups contributing to the content and format to be used. Alpha testing, involving a cohort of patients who have already undertaken TKA, will also ensure the suitability of the education materials for future patients undergoing TKA. The focus of the material will be specifically targeted at enabling patients to make an informed choice of what expectations they would like to be able to achieve following their TKA and that this expectation is realistic and likely to be achieved. While expectations fulfillment has consistently been associated with post-TKA satisfaction, we acknowledge that other factors may also impact patient satisfaction in our study cohort and, as such, are also measuring a number of clinical, demographic, and psychological patient features in our patient group to evaluate the impact of these covariates on satisfaction with postsurgical outcomes.

We have decided to adopt a mixed methods approach to characterize patient expectations and expectation fulfillment; however, for the purposes of powering our randomized controlled study, we have selected the HSS-TKR as our primary outcome measure. This measure was selected as it has been used to assess the efficacy of education interventions in other studies [35]. While the benefit of this approach enables quantification of expectation level and type to be derived from the provision of a closed-question format, it does limit the type of expectations being characterized to those provided in the survey. For this reason, we have also included a number of open-ended questions which enable the participants to define their own expectations and provide a more individualized approach to characterizing expectations.

Our TKA patient cohort is limited to patients in the private health sector, and we acknowledge that there may be differences in the demographics, clinical, and social features and health literacy of this patient group, compared with those in the public health sector, which may limit the translation of findings to a broader clinical group. In light of this, the findings from this study can be considered a pilot phase, which can inform future implementation of the educational materials developed in this study in a wider range of patients undergoing TKA. Moreover, we anticipate that it will be necessary for external validation of the findings for this study to be verified in a cohort of public patients prior to further implementation approaches being considered. In addition to ensuring equitable access to the educational materials developed, implementation strategies will need to consider the capacity for embedding materials into digital platforms accessible to the target patient population. Differences in cultural settings may also limit the generalizability of findings from this study. Previous studies conducted by our team [30] and findings by others [29] have shown that Australian patients undergoing TKA set high expectations and have goals to achieve high-impact physical activities following surgery, which indeed may not be reflected in other countries.

### Future Directions

The findings from this study will inform future approaches to accurately characterize patient expectations of postsurgical outcomes following TKA. This will enhance shared decision-making and extend a more patient-centric approach to measures of success following TKA.

Although the findings from this study may be viewed as a part of a pilot phase, they can serve as a foundation for developing future implementation strategies to scale the targeted education program across a larger clinical cohort.

### Conclusion

In conclusion, this study will develop innovative educational materials co-designed with key stakeholders to help patients undergoing TKA set realistic expectations for their postsurgical outcomes. By aligning expectations with achievable goals, the program aims to improve expectation fulfillment, enhance satisfaction, and empower patients. In addition, study findings will support shared decision-making practices and optimize rehabilitation programs, ensuring that patients receive the right care at the right time to achieve their individual goals.

## Supplementary material

10.2196/71898 Checklist 1 COREQ checklist.

## Abbreviations

DASS-21: 21-item Depression, Anxiety Stress Scale
DQO Index: Direct Questioning of Objectives Questionnaire Index
FJS: Forgotten Joint Score
HLS-EU-Q12: 12-item European Health Literacy Survey Questionnaire
HSS-TKR: Hospital for Special Surgery Total Knee Replacement Expectations Survey
KOOS-12: 12-item Knee Injury and Osteoarthritis Outcome Score
OKS: Oxford Knee Score
PEMAT-A/V: Patient Education Materials Assessment Tool—Audio Visual
REDCap: Research Electronic Data Capture
SCT: social cognitive theory
TKA: total knee arthroplasty
